# Repair of a Perforated Marginal Ulcer Seven Years After Roux-en Y Gastric Bypass: A Case Report and Review of Literature

**DOI:** 10.7759/cureus.40750

**Published:** 2023-06-21

**Authors:** Yesenia Brito, Omobolanle Kayode, Dominique Peters, Ameya Nair, Melyssa James, Frederick Tiesenga

**Affiliations:** 1 General Surgery, St. George’s University School of Medicine, True Blue, GRD; 2 General Surgery, Saint James School of Medicine, Arnos Vale, VCT; 3 General Surgery, West Suburban Medical Center, Chicago, USA

**Keywords:** bariatric complications, bariatric surgery, roux-en-y gastric bypass, gastrointestinal perforations, anastomotic ulcers, marginal ulcer

## Abstract

Marginal ulcers can be a rare but fatal post-surgical complication of Roux-en-Y gastric bypass (RYGB). In this report, we will describe the case of a 70-year-old female, with a seven-year status post-gastrojejunostomy who presented with a perforated marginal ulcer and showed significant improvement in her symptoms after a revisional operation for the marginal ulcer. The goal of this case report is to make clinicians aware of the unique complications of RYGB and outline the appropriate workup for patients presenting with post-bariatric abdominal pain.

## Introduction

Marginal ulcers, characterized by mucosal erosion at the gastrojejunal anastomosis, account for the majority of postoperative complications in up to 52% of patients who have undergone Roux-en-Y gastric bypass (RYGB) [1]. Although the exact causes of marginal ulcers are not fully understood, there are several risk factors that have been identified to be highly linked to its occurrence, with smoking and non-steroidal anti-inflammatory drugs (NSAIDs) making the top of the list [2]. Patients may present with a variety of upper gastrointestinal symptoms, including abdominal pain, nausea, vomiting, hematemesis, stromal obstruction, and perforation [1]. Medical and surgical management is required to prevent further complications and mortality.

The initial management of marginal ulcers consists of lifestyle modifications such as smoking cessation, discontinuation of NSAIDs, and proton pump inhibitor use (PPI). However, surgical intervention is required in complex ulcers, specifically, perforated, penetrated, obstructive, and bleeding marginal ulcers [1]. The ordinary approach for marginal ulcer resection involves excising the entire ulcer bed and restoring the anatomy through the creation of a new gastrojejunostomy. Surgery is also considered in simple marginal ulcers that are refractory to treatment [3].

A study by Patel et al. [4] found that out of 39 patients who needed revisional surgery due to marginal ulceration, 87% remained symptom-free following repair. Additionally, non-smokers with intractable marginal ulcers were found to have a better chance of symptom resolution than patients who smoked [4]. Furthermore, the rate of revision was notably lower following laparoscopic RYGB in comparison to open RYGB [4]. Open RYGB involves a large incision in the abdomen, providing direct visualization of the surgical field. Alternatively, laparoscopic RYGB entails several small incisions through which specialized instruments are inserted. This approach is considered minimally invasive, resulting in smaller scars, reduced postoperative pain, and faster recovery compared to open RYGB. The choice between the two techniques is determined by individual patient factors.

In this case report and literature review, we present the case of a 70-year-old female with a perforated marginal ulcer after RYGB who responded favorably to revisional operation.

## Case presentation

We present the case of a 70-year-old female with a body mass index (BMI) of 32.9 kg/m^2^. The patient’s past medical history was significant for obesity status post-ventral hernia, gastrojejunostomy, gastroesophageal reflux disease, peptic ulcer disease, *Helicobacter pylori* infection, diverticulosis, hyperplastic colon polyps, renal and hepatic cyst, schizophrenia, depression, and anxiety. The patient had a social history notable for 50 years of heavy alcohol consumption and a current smoking history of 7.5 pack years.

In 2016, the patient underwent gastrojejunostomy due to a bleeding giant peptic ulcer, with no postoperative complications other than an allergic reaction to the staples placed at the incision site.

The patient presented to the emergency department (ED) complaining of a month-long history of persistent non-radiating epigastric pain. The pain was associated with nausea, severe tarry stools, cough, and shortness of breath.

**Figure 1:**
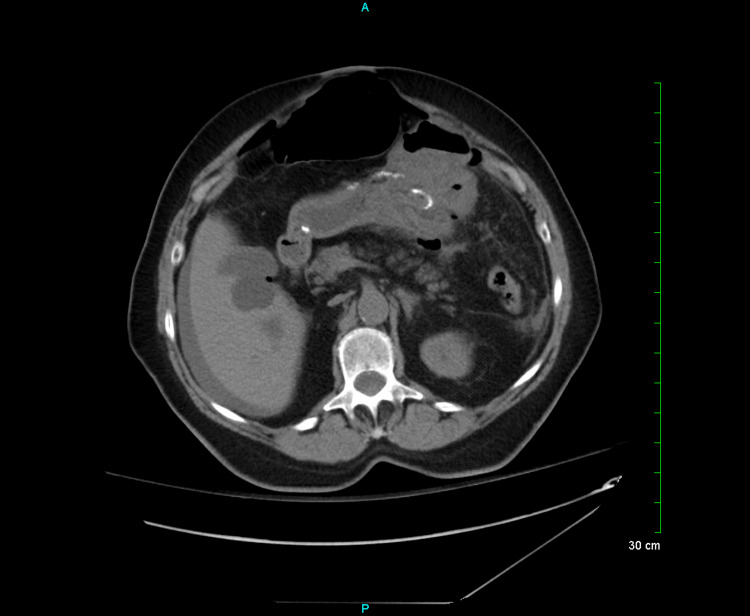
Gastrojejunostomy with wall thickening at the anastomotic site, probable ulceration, free intraperitoneal air, and fluid highly suspicious for a perforated marginal ulcer.

**Figure 2:**
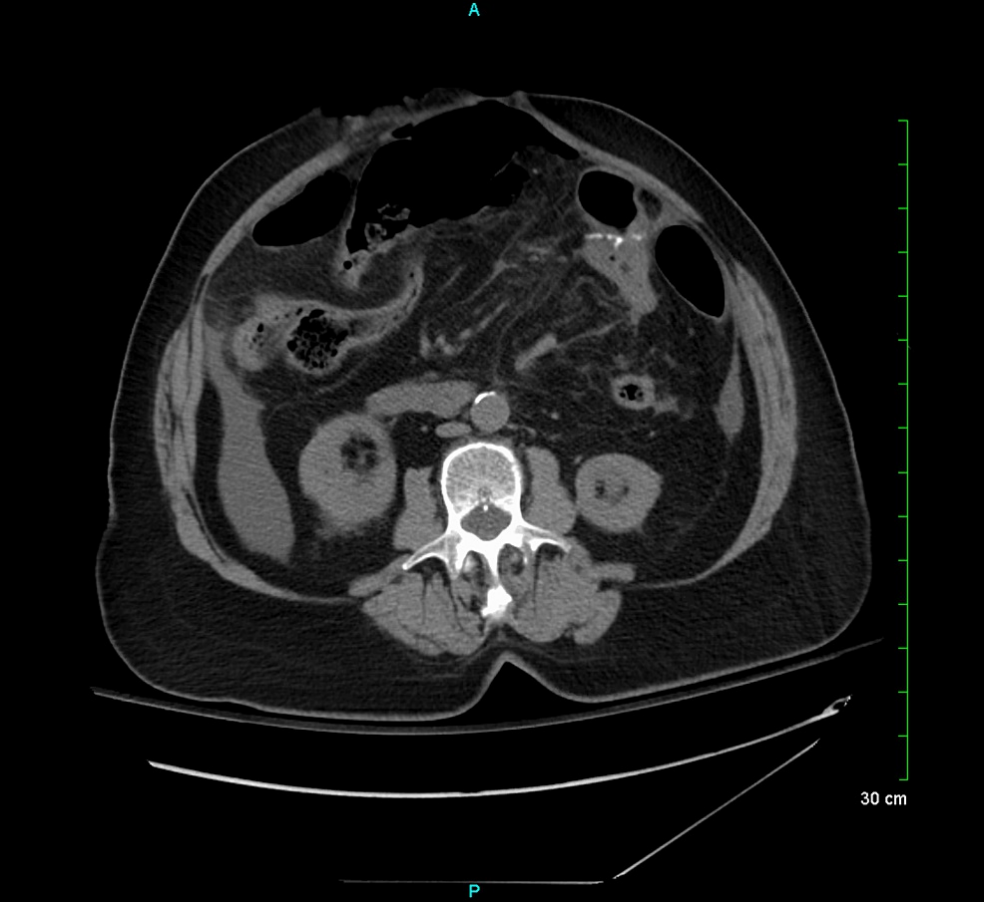
Anterior abdominal wall hernia without findings suggesting obstruction.

On admission, a computed tomography (CT) scan of the abdomen and pelvis without intravenous (IV) contrast demonstrated gastrojejunostomy with wall thickening at the anastomotic site (Figure 1) and anterior abdominal wall hernias (Figure 2).

The patient was taken to the operating room for an exploratory laparotomy. Upon entering the peritoneal cavity, a large amount of murky fluid was noted. The previous gastrojejunostomy was perforated at the level of the anastomosis feely leaking fluid into the abdominal cavity. The small bowel was irregular and hard at the level of the anastomosis. The entire anastomosis as well as the limb of the small bowel were resected. The specimen was sent to pathology. The Roux limb was oriented and a new side-to-side anastomosis was made between the stomach and the Roux limb. The anastomosis was checked under pressure and reinforced with sutures. A Jackson-Pratt drain was left in the area of the new gastrojejunostomy. The abdomen was then closed anatomically, repairing the incisional hernia at the level of the fascia.

**Figure 3:**
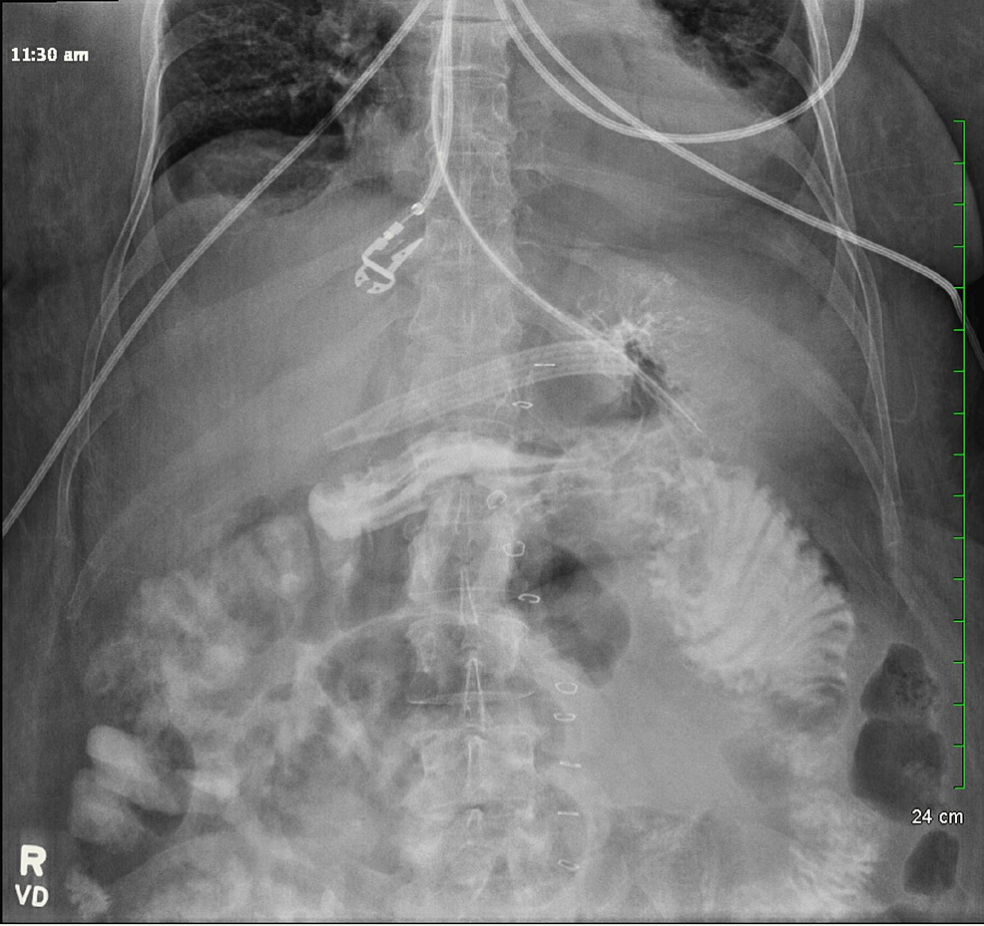
Upper gastrointestinal tract radiography with fluoroscopy showing contrast opacifying the stomach and flowing to the gastrojejunostomy without obstruction and extravasation.

An upper gastrointestinal study performed on postoperative day (POD) four showed oral contrast flowing easily into the Roux limb with no evidence of extravasation (Figure 3).

**Figure 4:**
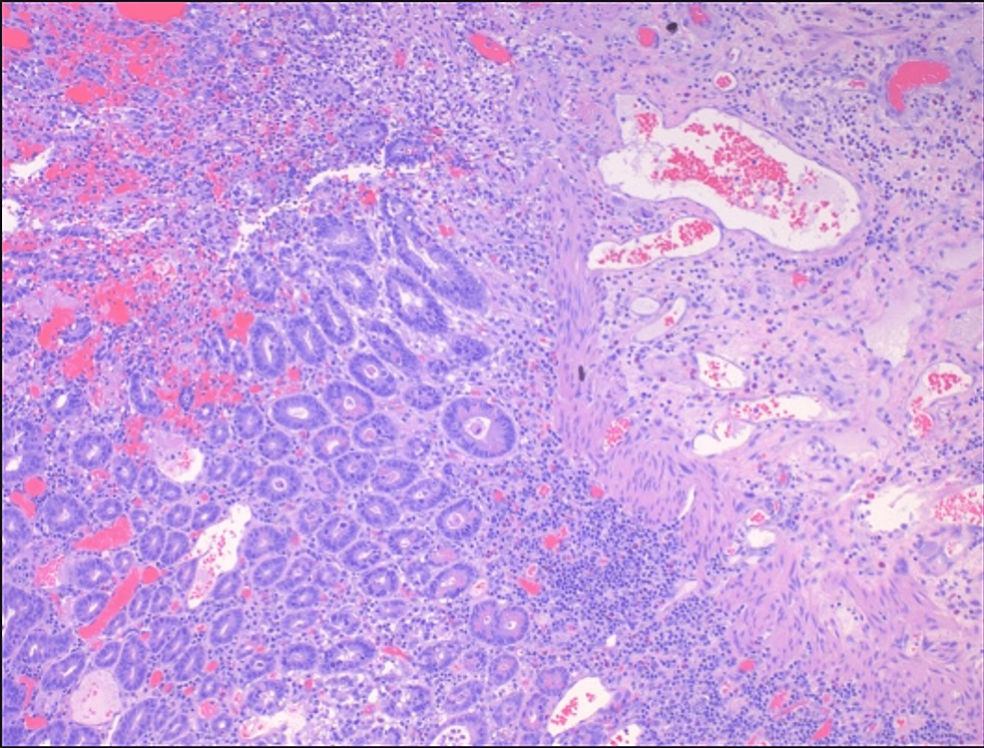
Acute and chronic inflammation, granulation tissue formation, ischemic, hemorrhagic, and reactive changes (hematoxylin and eosin stain).

The pathology report displayed perforation associated with ulcer, acute and chronic inflammation, granulation tissue formation, ischemic, hemorrhagic change, and reactive changes (Figure 4). The specimen was negative for dysplasia or malignancy. Hernia and umbilical sac specimens showed subcutaneous tissue with focal acute and chronic inflammation and reactive change.

The patient’s postoperative course was uneventful. The patient was advanced to a clear liquid diet on POD five which was tolerated well. The surgical team continued to monitor the patient for a week after the gastrojejunostomy. The patient reported no complaints of epigastric pain. The patient is being medically managed with PPIs, with a follow-up endoscopy planned.

## Discussion

Patients who have undergone gastric bypass in the past often present with several upper gastrointestinal complaints and symptoms. Patients who have developed marginal ulcers or stomal ulcers complain of epigastric pain that does not change in severity with food. Some patients also experience nausea, vomiting, hematemesis, and stomal obstruction [1,4]. Complaints of vague abdominal symptoms from patients following gastric bypass surgery deserve a full and thorough investigation [4].

Marginal ulcerations are one of the most complex postoperative complications following RYGB. This ulcer develops from mucosal erosion at the gastrojejunal anastomosis, most commonly on the jejunal side. There are many causes of marginal ulcers including poor tissue perfusion caused by tension or ischemia at the anastomosis, presence of non-absorbable sutures or staples, excess acid exposure in the gastric pouch due to gastro-gastric fistulas, NSAID use, *Helicobacter pylori* infection, and smoking [5].

Primarily, treatment for marginal ulcers is high-dose PPIs [6]. While there is no consensus on the dose or formulation of therapy, it has been recommended by some authors that to enhance the absorption in bypass patients, soluble PPIs should be administered. In addition to PPIs twice daily, it is recommended to add 1 gram of sucralfate four times daily for at least three months before considering surgical revision [6].

Medical management of marginal ulcers is successful in up to 95% of patients. Surgery may be indicated if marginal ulcers perforate or if persistent pain or recurrent bleeding occurs despite maximal medical therapy. In hemodynamically stable patients, revision operation of the gastrojejunostomy with truncal vagotomy should occur. In unstable patients, a Graham patch can be used to seal any perforation, the local area washed out, and a feeding tube placed. If stenosis occurs, the gastrojejunal anastomosis can be revised at a later time when the patient is more stable [7].

Research shows the incidence of postoperative RYGB with a complication of stomal ulcer to be 0.6-16%, with the actual incidence being much higher than reported [1]. The development of marginal ulcers is usually found where the gastric remnant is stapled but not divided [1]. In a study among 2,282 patients who underwent RYGB, it was proven that 122 developed marginal ulcers. Of the 122 patients, 39 underwent revision surgery due to treatment failure, and 34 out of those 39 patients reported complete symptom relief, indicating efficacy [4].

RYGB revision is typically indicated in cases where conservative management, such as lifestyle modification and medication, has failed to resolve the issue. Marginal ulceration, inadequate weight loss, stricture of gastrojejunal stroma, and BMI ≥40 kg/m^2^ or ≥35 kg/m^2^ with obesity-related comorbidity are features that make a patient a good candidate for this procedure [8]. Some contraindications include severe malnutrition, excessive adhesions, an enlarged fatty liver limiting the surgical field of view, and uncontrolled psychiatric illness. Our patient had the classical symptoms of RYGB complication, such as severe abdominal pain, nausea, and a history of prior RYGB. The confirmation of a perforated marginal ulcer on the CT scan made this patient undergo an emergent corrective procedure.

The vast majority of patients with marginal ulcers have a favorable prognosis. However, when this is not the case, surgical revision may be indicated with endoscopic follow-up. Complications of surgical revision include infection, postoperative bleeding, thrombi, and lengthy hospital stay [9]. Postoperatively, risk factors for the development of marginal ulcers, such as smoking and NSAID use, should be avoided to aid in appropriate outcomes [1]. Patients should be monitored and assessed for complications before advancing to a clear liquid diet and maintained on PPIs thereafter [1].

A perforated marginal ulcer is a rare but serious complication of RYGB, which requires prompt surveillance, diagnosis, and treatment to prevent morbidity and mortality. In this case, our patient who suffered from a perforated marginal ulcer post-RYGB benefitted immensely from the revisional Roux-en-Y gastrojejunostomy with a report of complete symptom resolution and an uneventful postoperative course. The management of marginal ulcers requires a multidisciplinary approach involving surgeons, gastroenterologists, and nutritionists to optimize patient outcomes and prevent future complications.

## Conclusions

Perforated marginal ulcers are a rare but serious complication of RYGB gastrojejunostomy procedures that can lead to life-threatening complications. Early diagnosis and prompt surgical interventions are critical in achieving good outcomes for these patients. Regular endoscopic surveillance and lifestyle modification of smoking and NSAID cessation should be recommended to prevent a recurrence. Prophylaxis such as PPIs should be considered with ongoing follow-up. In the surveillance of patients post-RYGB, it is imperative that clinicians are aware of the risk of developing rare complications and evaluate the need for emergent operative management.

## References

[1] Racu C, Mehran A (2010). Marginal ulcers after Roux-en-Y gastric bypass: pain for the patient…pain for the surgeon. Bariatric Times.

[2] Azagury DE, Abu Dayyeh BK, Greenwalt IT, Thompson CC (2011). Marginal ulceration after Roux-en-Y gastric bypass surgery: characteristics, risk factors, treatment, and outcomes. Endoscopy.

[3] Nguyen NT, Hinojosa MW, Gray J, Fayad C (2007). Reoperation for marginal ulceration. Surg Endosc.

[4] Patel RA, Brolin RE, Gandhi A (2009). Revisional operations for marginal ulcer after Roux-en-Y gastric bypass. Surg Obes Relat Dis.

[5] Sapala JA, Wood MH, Sapala MA, Flake TM Jr (1998). Marginal ulcer after gastric bypass: a prospective 3-year study of 173 patients. Obes Surg.

[6] Steinemann DC, Bueter M, Schiesser M, Amygdalos I, Clavien PA, Nocito A (2014). Management of anastomotic ulcers after Roux-en-Y gastric bypass: results of an international survey. Obes Surg.

[7] Sanyal AJ, Sugerman HJ, Kellum JM, Engle KM, Wolfe L (1992). Stomal complications of gastric bypass: incidence and outcome of therapy. Am J Gastroenterol.

[8] Fringeli Y, Worreth M, Langer I (2015). Gastrojejunal anastomosis complications and their management after laparoscopic Roux-en-Y gastric bypass. J Obes.

[9] Acquafresca PA, Palermo M, Rogula T, Duza GE, Serra E (2015). Early surgical complications after gastric by-pass: a literature review. Arq Bras Cir Dig.

